# Factors Associated with Hospital Length of Stay among Cancer Patients with Febrile Neutropenia

**DOI:** 10.1371/journal.pone.0108969

**Published:** 2014-10-06

**Authors:** Regis G. Rosa, Luciano Z. Goldani

**Affiliations:** Infectious Diseases Unit, Hospital de Clínicas de Porto Alegre, Universidade Federal do Rio Grande do Sul, Porto Alegre, Brazil; Federico II Ubiversity Of Naples, Italy

## Abstract

**Purpose:**

This study sought to evaluate factors associated with hospital length of stay in cancer patients with febrile neutropenia.

**Methods:**

A prospective cohort study was performed at a single tertiary referral hospital in southern Brazil from October 2009 to August 2011. All adult cancer patients with febrile neutropenia admitted to the hematology ward were evaluated. Stepwise random-effects negative binomial regression was performed to identify risk factors for prolonged length of hospital stay.

**Results:**

In total, 307 cases of febrile neutropenia were evaluated. The overall median length of hospital stay was 16 days (interquartile range 18 days). According to multiple negative binomial regression analysis, hematologic neoplasms (*P* = 0.003), high-dose chemotherapy regimens (*P*<0.001), duration of neutropenia (*P*<0.001), and bloodstream infection involving Gram-negative multi-drug-resistant bacteria (*P* = 0.003) were positively associated with prolonged hospital length of stay in patients with febrile neutropenia. The condition index showed no evidence of multi-collinearity effect among the independent variables.

**Conclusions:**

Hematologic neoplasms, high-dose chemotherapy regimens, prolonged periods of neutropenia, and bloodstream infection with Gram-negative multi-drug-resistant bacteria are predictors of prolonged length hospital of stay among adult cancer patients with febrile neutropenia.

## Introduction

Febrile neutropenia (FN) is a common complication of cancer treatment. The absolute neutropenia caused by intensive cytotoxic chemotherapy increases the risk of severe infections, which frequently require hospitalization for administration of broad-spectrum antibiotics to minimize morbidity and mortality [1], [2].

Hospital length of stay (LOS) is an important marker of clinical severity and use of resources in the context of FN [3]. Neutropenic cancer patients who require prolonged LOS are at increased risk of multi-drug-resistant (MDR) infections and delays in their antineoplastic treatments [4], [5], which can, in turn, have implications for cancer treatment outcomes. Moreover, given that diagnostic and treatment procedures in patients with FN are often associated with large financial expenditures, prolonged LOS has a negative impact on healthcare resource use and costs [6], [7]. In specialized centers, the median cost of hospitalization per episode of FN may be as high as $24,000 USD [3] with an attributable cost excess greater than $12,000 USD [8].

Understanding the factors that prolong LOS in patients with FN may improve our ability to reduce costs and improve their quality of care. Therefore, we performed this study with the aim of evaluating the factors associated with increased LOS in hospitalized adult cancer patients with FN.

## Patients and Methods

### Study design, patients, and settings

A prospective cohort study was conducted in the hematology ward of the Hospital de Clínicas de Porto Alegre, Rio Grande do Sul, a tertiary referral center for bone marrow transplantation in southern Brazil. All patients admitted between October 2009 and August 2011 and fitting the eligibility criteria were enrolled in this study. Inclusion criteria comprised age ≥18 years, neutropenia (absolute neutrophil count <500 cells/mm^3^ or <1000 cells/mm^3^ with an expectation of a decrease to <500 cells/mm^3^ during the ensuing 48 h), and fever (a single axillary temperature measurement ≥38.5°C or ≥38.0°C sustained for 1 h). Exclusion criteria comprised individuals receiving only palliative treatment, an indication for outpatient treatment, or neutropenia caused by something other than manifestations of hematological malignancy, bone marrow or peripheral blood stem cell transplantation, or adverse reaction to chemotherapy. Subjects were eligible to re-enter the study with a second or subsequent episode of FN if they had been discharged from hospital after completing treatment for a prior episode of FN.

### Treatment protocol

Febrile neutropenic patients were treated according to the 2010 updated guidelines of the Infectious Diseases Society of America [9]. Initial antimicrobial treatment was β-lactam monotherapy with anti-pseudomonal activity (i.e., cefepime, piperacillin–tazobactam, or a carbapenem); a glycopeptide (i.e., vancomycin) was added to this initial empiric regimen only in patients with hemodynamic instability, suspected catheter-related infection, or infection of skin and soft tissue. Empiric antifungal therapy with amphotericin B deoxycholate was administered in patients with persistent fever after 4 days of treatment with broad-spectrum antibiotics. No patients received antibacterial prophylaxis. Antifungal prophylaxis with fluconazole was routinely administered to patients in whom the anticipated duration of neutropenia was>7 days. Acyclovir antiviral prophylaxis was administered to herpes simplex virus seropositive patients undergoing allogeneic hematopoietic stem-cell transplantation or leukemia induction chemotherapy.

### Independent variables

The independent variables to be examined in this study were selected based on previously reported associations with prognosis in patients with FN [10]–[13]. All baseline characteristics were verified at the onset of fever by a medical research team not associated with patient care. Clinical comorbidity was defined as the presence of heart failure, diabetes mellitus, chronic pulmonary disease, chronic liver disease, or chronic renal failure. The patients were allocated to two groups based on their chemotherapy regimens: a high-dose chemotherapy group that included patients undergoing hematopoietic stem cell transplantation or induction chemotherapy and a standard-dose chemotherapy group that included patients undergoing consolidation or maintenance chemotherapy. Profound neutropenia was defined as an absolute neutrophil count <100 cells/mm^3^ at the onset of FN. Prolonged neutropenia was defined as duration of neutropenia>7 days after the onset of FN. Nosocomial-acquired FN was defined as FN developing 48 h or more after hospitalization. Microbiological studies were performed at the onset of fever according to standard practice and included two separate blood samples from two different sites. In the absence of an indwelling central venous catheter, these blood sets were obtained from two distinct peripheral veins. When an indwelling central venous catheter was present, one sample for blood culture was obtained through that catheter and the other from a peripheral vein. The susceptibilities of the isolated pathogens to antibiotics were evaluated according to the recommendations of the Clinical and Laboratory Standards Institute [14]. Polymicrobial bloodstream infection was defined as a bacteremic episode in which at least two different pathogens were isolated from the same blood sample. For Gram-positive bacteria, MDR bacteremia was defined as bloodstream infection (BSI) with methicillin-resistant staphylococci or vancomycin-resistant enterococci, whereas for Gram-negative bacteria it was defined as resistance to three or more classes of antimicrobial agents. Proven and probable invasive fungal infections (IFIs) were defined according to the criteria of the European Organization for Research and Treatment of Cancer-Invasive Fungal Infections Cooperative Group [15].

### Outcome and follow-up

The primary outcome of the present study was the LOS after the onset of FN. Patient follow-up was performed by researchers who were not associated with the assistant physician's team through interviews and medical record reviews using a standardized data collection instrument. Follow-up was maintained throughout each hospitalization.

### Statistical analysis

Independence could not be assumed because some patients had more than one episode of FN and were therefore evaluated more than once. Accordingly, stepwise random-effects negative binomial regression analysis was performed: this is a validated strategy for dealing with clustered data (that is, when observations in one cluster tend to be more similar to each other than to individuals in the rest of sample) [16]. All clinical and microbiological variables with a *P* value <0.10 in the univariate analysis were included. In the multivariate model, independent variables were eliminated from the highest to the lowest *P* value but remained in the model if the *P* value was <0.05. Incidence rate ratios (IRR) were estimated with 95% confidence intervals (95% CI). Multi-collinearity was assessed according to the condition index of the multivariate model: a condition index <10 denotes weak collinearity, 10–30 denotes moderate collinearity, and>30 denotes strong collinearity [17]. The statistical analysis was performed using STATA version 12 (Stata Corp LP, USA).

### Ethical considerations

Written informed consent was obtained from all study participants. The institutional review board of Hospital de Clínicas de Porto Alegre approved the study protocol and consent form (GPPG 09282). A copy of each written consent obtained is available for review from the Editor-in-Chief of this journal if necessary.

## Results

Three hundred and seven cases of FN (in 169 patients) were evaluated during the study period. Seventy-one patients (42% of the study cohort) had two or more episodes of FN; the maximum number of episodes in an individual patient was four. Relevant characteristics of all episodes of FN are shown in Table 1. Most of the cancers were hematological malignancies (78.8%); the most common being acute myeloid leukemia (48.5%), lymphoma (16.6%), and acute lymphoblastic leukemia (14.6%); in 53.4% of the cases, high-dose chemotherapy regimens were being administered.

**Table 1:** Clinical characteristics in 307 cases of febrile neutropenia.

Age, mean years ± SD	40.7±14.2
Female sex	148 (48.2)
Type of cancer	
Acute myeloid leukemia	149 (48.5)
Acute lymphoblastic leukemia	45 (14.6)
Chronic myeloid leukemia	18 (5.8)
Multiple myeloma	30 (9.7)
Lymphoma	51 (16.6)
Other solid tumors	14 (4.5)
Relapsing underlying disease	155 (50.4)
Clinical comorbidity	76 (24.7)
Phase of chemotherapy	
Induction	76 (24.7)
Consolidation	86 (28.0)
Maintenance	57 (18.6)
HSCT	88 (28.7)
ANC at the time of diagnosis of FN, median cells/mm^3^ (IQR)	130 (260)
Duration of neutropenia, median days (IQR)	9 (12)
Nosocomial-acquired episodes of FN	250 (81.7)
Bloodstream infection	115 (37.4)
BSI involving Gram-positive bacteria	46 (14.9)
BSI involving Gram-negative bacteria	74 (24.1)
Polymicrobial BSI	12 (3.9)
BSI involving Gram-positive MDR bacteria	27 (8.7)
BSI involving Gram-negative MDR bacteria	12 (3.9)
Proven or probable IFI	22 (7.1)

Data presented as n (%) unless otherwise indicated. SD  =  standard deviation; HSCT  =  hematopoietic stem cell transplantation; ANC  =  absolute neutrophil count; FN  =  febrile neutropenia; IQR  =  interquartile range (P75–P25); BSI  =  bloodstream infection; MDR  =  multi-drug-resistant; IFI  =  invasive fungal infection.

During the study period, 115 BSIs were documented. The predominant isolates from blood were *Escherichia coli* (41.7%), coagulase-negative staphylococci (31.3%), *Klebsiella pneumoniae* (11.3%), *Pseudomonas aeruginosa* (9.5%), viridans streptococci (6.9%), and *Enterococcus* spp (3.4%). Among all BSIs evaluated, 38 episodes (33.0%) were caused by MDR bacteria; of these, 68.4% were caused by Gram-positive bacteria, 29.0% by Gram-negative bacteria, and 2.6% by both Gram-positive and Gram-negative bacteria. Methicillin resistance and production of extended-spectrum beta-lactamase were the most frequent types of antimicrobial resistance, occurring in 96.2% of BSIs involving Gram-positive MDR bacteria and 83.3% of BSIs involving Gram-negative MDR bacteria. The overall *in vitro* rate of resistance of blood isolates to the initial antibiotics administered was 12.7%. The incidence of proven or probable IFI was 7.1%.

**Figure 1:**
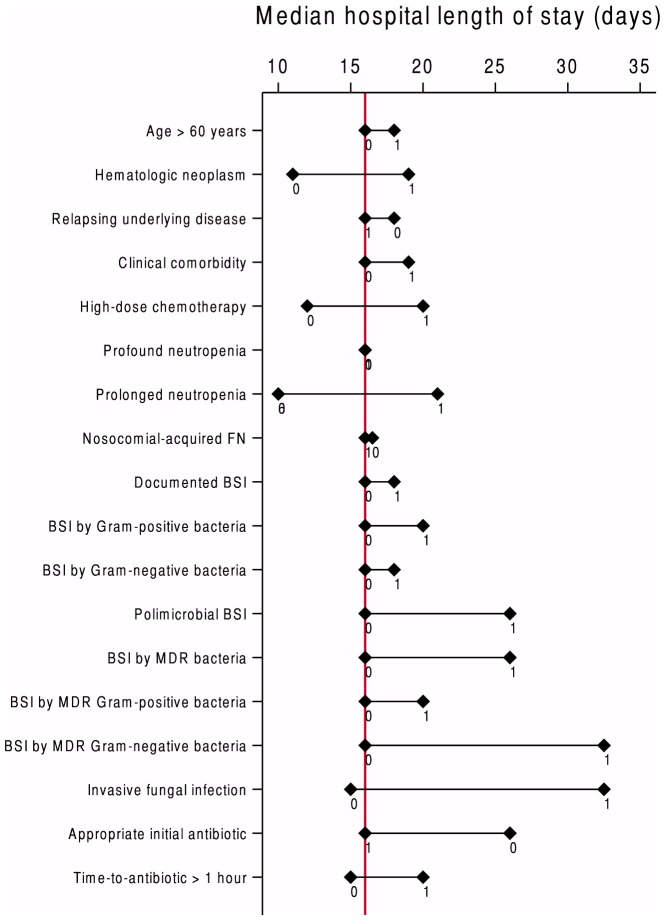
Median hospital length of stay among cases of febrile neutropenia according to clinical characteristics. The red line represents the median length of hospital stay of the entire cohort. For each variable, 1 and 0 represent, respectively, the median LOS for cases with and without the clinical characteristic described in the corresponding row; BSI  =  bloodstream infection; MDR  =  multi-drug-resistant.

The median LOS of the all episodes of FN was 16 days (interquartile range [IQR] 18 days). Sixty-nine percent of the cases were hospitalized for longer than 10 days. The median LOS for those admitted for 10 days or less was 8 days (IQR 3 days). The median LOS for those admitted for longer than 10 days was 22 days (IQR 17 days). The median LOS according to case characteristics are shown in Figure 1; the greatest differences in median LOS according to the presence or absence of certain clinical features were found in the following categories: IFI, BSI involving Gram-negative MDR bacteria, and prolonged neutropenia.

According to the univariate analysis (Table 2), hematologic neoplasms (*P*<0.001), high-dose chemotherapy regimens (*P*<0.001), duration of neutropenia (*P*<0.001), MDR BSI (*P* = 0.04), BSI involving Gram-negative MDR bacteria (*P* = 0.009), and proven or probable IFI (*P* = 0.002) were statistically associated with LOS. Interestingly, variables related to antimicrobial treatment, such as *in vitro* sensitivity of blood isolates to initial antibiotic treatment (*P* = 0.07) and time to initial antibiotic (*P* = 0.24) were not significant predictors of LOS. According to multivariate analysis (Table 2), hematologic neoplasms (*P* = 0.003), treatment with high-dose chemotherapy regimens (*P*<0.001), duration of neutropenia (*P*<0.001), and BSI by MDR Gram-negative bacteria (*P* = 0.006) were positively associated with LOS. The condition index of the final multivariate negative binomial regression was 5.5, indicating little collinearity among the explanatory variables in the model. In cases with hematologic neoplasms and those receiving high-dose chemotherapy before the episode of FN, the median LOS was 30% and 46%, respectively, longer than in cases without these risk factors. Each additional day of neutropenia was associated with a 2% increase in the total LOS. In cases with BSI caused by MDR Gram-negative bacteria, the median LOS was 62% longer than in other patients.

**Table 2:** Negative binomial regression of factors associated with hospital length of stay among cases of febrile neutropenia in cancer patients.

Variables	Univariate analysis		Multivariate analysis	
	IRR (95% CI)	*P* value	IRR (95% CI)	*P* value
Age, years	0.99 (0.98–1.00)	0.10	-	-
Hematologic neoplasm	1.64 (1.35–2.00)	<0.001	1.30 (1.09–1.55)	0.003
Relapsing underlying disease status	0.87 (0.74–1.03)	0.11	-	-
Clinical comorbidity	1.02 (0.84–1.23)	0.79	-	-
High-dose chemotherapy regimens	1.48 (1.26–1.73)	<0.001	1.43 (1.24-1.64)	<0.001
ANC at the time of the diagnosis of FN, cells/mm^3^	1.0003 (0.99–1.00)	0.05	-	-
Duration of neutropenia, median days	1.02 (1.01–1.03)	<0.001	1.02 (1.01-1.02)	<0.001
Nosocomial-acquired episode of FN	0.91 (0.74–1.12)	0.40	-	-
Bloodstream infection	1.007 (0.85–1.19	0.93	-	-
BSI involving Gram-positive bacteria	1.08 (0.86–1.36)	0.47	-	-
BSI involving Gram-negative bacteria	1.009 (0.83–1.22)	0.92	-	-
Polymicrobial BSI	1.46 (0.97–2.21)	0.06	-	-
MDR BSI	1.28 (1.00–1.63)	0.04	-	-
BSI involving Gram-positive MDR bacteria	1.09 (0.82–1.46)	0.51	-	-
BSI involving Gram-negative MDR bacteria	1.72 (1.14–2.58)	0.009	1.62 (1.15–2.29)	0.006
Proven or probable IFI	1.63 (1.20–2.22)	0.002	-	-
*In vitro* sensitivity of blood isolates to initial antibiotic treatment	0.79 (0.62–1.01)	0.07	-	-
Time to initial antibiotic, hours	1.01 (0.98–1.04)	0.24	-	-

Note. The incidence rate ratio (IRR) represents the change in the dependent variable (days of hospitalization) in terms of percentage (determined by the amount the IRR is above or below 1) per unit increase of continuous independent variables or in the yes versus no group for binary independent variables. ANC  =  absolute neutrophil count; FN  =  febrile neutropenia; BSI  =  bloodstream infection; MDR  =  multi-drug-resistant; IFI  =  invasive fungal infection.

## Discussion

In the present cohort of patients with one or more episodes of FN, hematologic neoplasms, high-dose chemotherapy regimens, duration of neutropenia, and BSI with Gram-negative MDR bacteria were positively associated with prolonged LOS among hospitalized adult cancer patients with FN.

Reported median LOS in the context of FN varies according to the category of patient studied. In the study by Kuderer et al., LOS among cancer patients with FN had a range from 8.1 days (for patients with solid tumors) to 19.7 days (for patients with leukemia) [18]. Basu et al. reported a median LOS of 5 days in pediatric cancer patients with both high- and low-risk episodes of FN; specifically, the median LOS for patients admitted for longer than 5 days was 12 days [19]. In addition, Weycker et al. reported a mean LOS of 8.4 days for adult patients with FN who were receiving myelosuppressive chemotherapy for solid tumors or non-Hodgkin lymphomas [20]. The prolonged median LOS found in our study (16 days) is consistent with the characteristics of our study sample, which included a large proportion of high-risk hematologic patients receiving high-dose chemotherapy regimens. Moreover, we evaluated only patients receiving intravenous chemotherapy; this fact also contributed to the prolonged LOS seen in our study population.

The factors we identified as associated with LOS are supported by findings of previous studies. Consistent with our findings, Haeusler et al. [21] showed a correlation between MDR Gram-negative bacteremia and prolonged hospital and intensive care unit length of stay in pediatric oncology patients. Interestingly, the relationship between BSI with MDR bacteria and LOS can become a vicious cycle because these two variables are reciprocal risk factors: prolonged LOS increases the risk of MDR bacteremia, which in turn increases the risk of prolonged hospitalization. Hematological malignancies are also reportedly risk factors for complications during episodes of FN; Taccone et al. [22] showed that, in an intensive care setting, patients with hematological cancer were more seriously ill and more commonly had sepsis, acute respiratory distress syndrome, and renal failure than did patients with solid cancers. The impact of prolonged neutropenia caused by high-dose chemotherapeutic regimens on LOS has also been well documented. Previously validated strategies have focused on reducing the duration of neutropenia through interventions such as prophylactic administration of granulocyte colony-stimulating factors, which is often associated with a decrease in the incidence of infections and shorter hospitalizations in neutropenic patients, without an impact on mortality [23]–[25].

The major limitation of our study is the observational design; we cannot be certain that we have identified all potential confounding factors. However, both assessment of independent variables by a research group not involved in patient care and the use of a prospective design with an objective endpoint contributed to the methodological strength of this study.

Identifying risk factors for prolonged LOS in patients with FN is of paramount importance to clinicians and healthcare administrators: this knowledge may guide preventative measures focused on decreasing LOS to both improve care (avoiding nosocomial infections and delays in cancer treatment) and optimize resource consumption (the duration of hospital stay is directly related to cost). Future trials are needed to evaluate the impact on LOS of directed measures focused on prevention and management of the factors associated with prolonged LOS among cancer patients with FN.
